# Chicago Society of Physicians and Surgeons

**Published:** 1874-07

**Authors:** John Bartlett

**Affiliations:** President, in the Chair


***Transactions of the Chicago Society of Physicians and Surgeons.*** *Regular Meeting, May* 25, 1874. Dr. JOHN BARTLETT, *President, in the Chair. Reported by* RALPH E. STARKWEATHER, M.D.

The Society met as usual at the Grand Pacific Hotel, and on motion of Dr. Owens, the minutes of the previous meeting were approved.

Dr. Trimble was prepared to submit only a partial report from the Board of Censors, and requested that hereafter the members of the Society would comply more strictly with the third article of the constitution in reference to giving full information respecting the candidates proposed for admission into the Society.

Doctors J. W. Freer, W. A. Harvey and H. Hooper were unanimously elected members.

The names of Doctors H. M. Bannister, H. W. Jones, M. O. Heydock, and H. W. Boyd, were proposed, and referred to the Censors.

The customary order of proceedings was now, on motion, suspended, by the presentation and discussion of a series of resolutions and amendments, which will await final action at subsequent meetings.

Dr. Trimble proposed, as an addition to the first article of the constitution, to insert "are now or have hitherto been regular practitioners in the city of Chicago, and are not now engaged in other business." This was further amended by Dr. Owens, seconded by Dr. Hyde, "that no applicants be considered eligible as members of this Society who have not been engaged in practice three years.

Dr. F. H. Davis thought that the period of three years was rather too long, but would favor an interval of one year, after graduation. It is really the young men who do much of original investigation and study, before they become much engaged in business; and it would discourage such men were they kept out of societies, unable to present the results of their studies.

Dr. Owens. The limit of three years is approved by many useful members of this Society not here present this evening.

Dr. Wood would like to see the period made three, or even seven years, as it would elevate the standard of the Society, and make it compare favorably with those of other countries and cities.

Drs. W. C. Lyman and Blake thought the clause relative to following any other business ought to be excised. After remarks by Doctors Henrotin and Jackson, Dr. Evans suggested that the rules of the Association and the powers of the Censors covered the subject already.

Dr. Hyde replied that the code of ethics of the American Medical Association does not prescribe who shall be its members, whether a druggist or physician; it simply directs physicians to discourage the selling by druggists of nostrums, patent medicines and the like.

Dr. Merriman opposed frequent changes in the constitution, as unnecessary. The Society could supplement the action of its Board of Censors when voting upon membership.

Dr. Hyde objected to the annual overflow of graduates from the colleges pouring into the Societies. He would favor the period of two years as a compromise.

Dr. Blake. It seems to me that it ought to be well understood that this Society does not admit a person who is not a practicing physician, or who may be a druggist; it is unfair to leave such responsibility to the Censors; they deserve to have our support.

Dr. Trimble said the amendment he offered had no intention of interfering with the private business of the members, except so far as the subject involved the drug business.

Dr. Jackson suggested adding to article 1, "who possess a good moral and professional reputation; who shall have been practitioners of medicine for two years; and who shall not be interested in the sale of drugs, medicines or surgical appliances."

On motion, the following resolutions were adopted:

WHEREAS, In the judgment of this Society, it is unbecoming for a member of the medical profession to engage in the business of druggist or apothecary; and

WHEREAS, It is reported that certain members of this Society have already, or are about to assume a financial interest in the prosecution of the general business of the sale of drugs and medicines; therefore,

*Resolved*, That the Censors of the Society are hereby requested to ascertain if such reports implicate any of its members; and if yea to any of such members, to report the fact of such traffic or business. And if these allegations be true, they shall be informed by the Censors that they will be allowed to resign honorably to themselves, during a period of one month from the date of such inquiries; and

*Resolved*, That in case any member who has been found to be engaged or interested financially in the business described, do not confess to the same, or refuse to resign the membership in one month, the Censors shall forthwith proceed to bring such member to trial, in the form and manner prescribed by the constitution and by-laws of this Society.

Dr. Hyde, in behalf of Dr. F. H. Davis, presented the report of the Banquet Committee, with vouchers, whieh was accepted, and a vote of thanks to the Committee carried.

It was voted that delegates from this Society to the Illinois State Society, and American Medical Association, be elected at the annual meeting.

The President announced the following appointments to the Sections:

1. On the Practice of Medicine—To report July 13, 1874: Dr. Walter Hay, Chairman; Drs. J. H. Hollister, W. C. Lyman, W. M. Boyd.

2. On Materia Medica and Therapeutics—Dr. D. B. Trimble, Chairman; Drs. J. H. Etheridge, F. H. Davis, H. P. Merriman.

3. Section on Surgery—Dr. E. Andrews, Chairman; Drs. E. Powell, H. McKennan, C. T. Parkes.

4. General Pathology—To report January 11, 1875: Dr. I. N. Danforth, Chairman; Drs. Lester Curtis, C. P. Simon, F. Henrotin, Jr.

5. Obstetrics and Diseases of Women—Dr. DeLaskie Miller, Chairman; Drs. A. Reeves Jackson, A. P. Peck, M. W. Wood.

On motion, the Society adjourned.

